# Oncotype DX recurrence score implications for disparities in chemotherapy and breast cancer mortality in Georgia

**DOI:** 10.1038/s41523-019-0129-3

**Published:** 2019-09-26

**Authors:** Lindsay J. Collin, Ming Yan, Renjian Jiang, Kevin C. Ward, Brittany Crawford, Mylin A. Torres, Keerthi Gogineni, Preeti D. Subhedar, Samantha Puvanesarajah, Mia M. Gaudet, Lauren E. McCullough

**Affiliations:** 1 Department of Epidemiology, Emory University Atlanta, Atlanta, GA 30322 USA; 2 Glenn Family Breast Center, Winship Cancer Institute, Emory University, Atlanta, GA 30322 USA; 3 Department of Epidemiology and Biostatistics, University of South Carolina, Columbia, SC 29208 USA; 4 Department of Radiation Oncology, Emory University School of Medicine, Atlanta, GA 30322 USA; 5 Department of Hematology and Medical Oncology, Emory University School of Medicine, Atlanta, GA 30322 USA; 6 Department of Surgery, Emory University School of Medicine, Atlanta, GA 30322 USA; 7 American Cancer Society, Atlanta, GA 30303 USA

**Keywords:** Prognostic markers, Predictive markers

## Abstract

Among women diagnosed with stage I–IIIa, node-negative, hormone receptor (HR)-positive breast cancer (BC), Oncotype DX recurrence scores (ODX RS) inform chemotherapy treatment decisions. Differences in recurrence scores or testing may contribute to racial disparities in BC mortality among women with HR+ tumors. We identified 12,081 non-Hispanic White (NHW) and non-Hispanic Black (NHB) BC patients in Georgia (2010–2014), eligible to receive an ODX RS. Logistic regression was used to estimate the odds of chemotherapy receipt by race and ODX RS. Cox proportional hazard regression was used to calculate the hazard ratios (HRs) comparing BC mortality rates by race and recurrence score. Receipt of Oncotype testing was consistent between NHB and NHW women. Receipt of chemotherapy was generally comparable within strata of ODX RS—although NHB women with low scores were slightly more likely to receive chemotherapy (OR = 1.16, 95% CI 0.77, 1.75), and NHB women with high scores less likely to receive chemotherapy (OR = 0.77, 95% CI 0.48, 1.24), than NHW counterparts. NHB women with a low recurrence score had the largest hazard of BC mortality (HR = 2.47 95% CI 1.22, 4.99) compared to NHW women. Our data suggest that additional tumor heterogeneity, or other downstream treatment factors, not captured by ODX, may be drivers of racial disparities in HR+ BC.

## Introduction

Gene expression profiles are powerful prognostic and predictive markers to guide treatment decisions among women diagnosed with breast cancer.^1,2^ More than two-thirds of all breast cancer diagnoses include tumors that are estrogen receptor (ER)-positive, human epidermal growth factor receptor-2 (HER2)-negative.^3^ Treatment for these tumors has historically included surgery followed by adjuvant systemic therapy, which includes endocrine therapy with or without chemotherapy.^4^ Chemotherapy is often accompanied by negative short and long-term side effects, which can impede on quality of life and activities of daily living.^5,6^ The Oncotype DX recurrence score (ODX RS) was originally validated among women diagnosed with ER+/HER2−, lymph node-negative breast cancer to guide adjuvant chemotherapy treatment decisions.^7^ Indications for Oncotype DX have changed over time as results from the *Trial Assigning Individualized Options for Treatment* (TAILORx) has demonstrated that chemotherapy may not be a necessary systemic treatment to prevent recurrence for women diagnosed with breast cancer with a low or intermediate ODX RS.^8,9^ Moreover, these indications may vary depending on menopausal status and lymph node involvement, as more recent evidence suggests that the recurrence score may be useful among postmenopausal women with limited lymph node involvement (1–3 positive nodes),^10–12^ which is being evaluated in the ongoing *Rx for Positive Node, Endocrine Responsive Breast Cancer* (RxPONDER) trial.^13^

Although advances in breast cancer diagnosis and treatment have led to an average 5-year survival of approximately 90% among non-metastatic breast cancer patients, disparities in health-care quality and access persist, affecting breast cancer outcomes in vulnerable populations.^14–18^ Since 2005, there has been a rapid uptake in the use of the 21-gene ODX RS to guide clinical treatment decisions. However, this increase has not been equitable across all breast cancer patients. Previous studies have reported variation in its application by age, socioeconomic status, and race, whereby younger breast cancer patients, higher income individuals, and non-Hispanic white (NHW) women are more likely to receive testing than other demographic subgroups.^19–23^

Racial disparities in breast cancer outcomes in the US are well-documented, with non-Hispanic black (NHB) women more likely to die from breast cancer than their white counterparts. Consistent with studies in other geographical areas,^24–26^ we observed that in the metropolitan Atlanta area, racial disparities in breast cancer mortality were more robust among women diagnosed with more favorable prognostic tumors that are known to have highly effective adjuvant therapies. Yet the mechanism of the disparity remains unresolved but may be due, in part, to the underlying genomic heterogeneity, whereby NHB women are more likely to be diagnosed with tumors with greater intratumoral heterogeneity captured by gene expression patterns.^27–29^ ODX RS may help to identify more aggressive tumors, further elucidating the underlying mechanism of this disparity in stage I–IIIa, hormone receptor (HR) positive tumors. In this study we aimed to further explore these seemingly paradoxical findings among early stage, HR+ tumors by (1) describing differences in Oncotype DX testing by race and patient characteristics, (2) describing the distribution of ODX RS of NHB and NHW women, (3) examining differences in receipt of chemotherapy by ODX RS between NHB and NHW women, and (4) examining differences in breast-cancer-specific mortality by ODX RS between NHB and NHW women diagnosed with breast cancer in Georgia.

## Results

### Study population

We identified 12,081 women eligible to receive an ODX RS in the Georgia Cancer Registry (GCR). Of the 12,081 women eligible to receive on ODX RS, 1332 NHB (47%) and 4418 NHW (48%) women diagnosed with a first primary breast cancer between 2010 and 2014 in Georgia, received an ODX RS (Table 1).^30^ Although NHB women were slightly less likely to receive an ODX RS compared to NHW women, we did not observe any meaningful racial disparities in the proportion of women receiving an ODX RS by tumor or patient characteristics. For both NHW and NHB breast cancer patients, women were more likely to have an ODX RS if they were 50–65 years of age and had private insurance at the time of diagnosis. Both NHW and NHB women were less likely to receive an Onocotype Dx recurrence score if they were over 65 years of age, had Medicare health insurance at the time of diagnosis, and lived in a lower socioeconomic status neighbourhood. The proportion of women receiving an ODX RS increased slightly over time; however, this uptake appeared to have a 1-year lag among NHB women.

**Table 1:** Patient demographic and clinicopathological characteristics among non-Hispanic White (NHW) and non-Hispanic Black (NHB) women diagnosed with breast cancer in Georgia (2010–2014) and eligible to receive an Oncotype DX (ODX) recurrence score^a^

Patient characteristics	NHW				NHB			
	Oncotype DX score received				Oncotype DX score received			
	Yes		No		Yes		No	
	*N*	%	*N*	%	*N*	%	*N*	%
Overall	4418	48	4818	52	1332	47	1513	53
ODX RS Group								
1	2580	58			686	52		
2	1469	33			456	34		
3	369	8.4			190	14		
Stage								
1	3258	35	3177	34	932	33	824	29
2	1146	12	1495	16	394	14	612	22
3	14	0.2	146	1.6	6	0.2	77	2.7
Grade								
1	1467	16	1786	20	341	12	395	14
2	2218	25	2007	22	682	25	596	22
3+	671	7.4	872	9.7	289	10	466	17
Missing	62		153		20		56	
Lymph Nodes								
Negative	3982	43	3891	42	1199	42	1187	42
1–3+	436	4.7	927	10	133	4.7	326	11
Chemotherapy								
Yes	867	9.5	1235	14	356	13	600	22
No	3468	38	3521	38	948	34	877	32
Missing	83		62		28		36	
Age								
<40	124	1.3	85	0.9	68	2.4	69	2.4
40–49	708	7.8	405	4.4	245	8.6	201	7.1
50–65	2220	24	1748	19	678	24	657	23
>65	1366	15	2580	28	341	12	586	21
Year of Dx								
2010	722	7.8	950	10	201	7.1	276	9.7
2011	895	9.7	972	11	222	7.8	274	9.6
2012	923	10	984	11	273	9.6	299	11
2013	948	10	933	10	307	11	352	12
2014	930	10	979	11	329	12	312	11
Insurance status								
Uninsured	44	0.5	43	0.5	24	0.8	39	1.4
Private	2658	29	1960	21	729	26	667	23
Medicaid	164	1.8	195	2.1	158	5.6	173	6.1
Medicare	1407	15	2482	27	358	13	578	20
Military	90	1.0	82	0.9	40	1.4	39	1.4
Unknown	55	0.6	56	0.6	23	0.8	17	0.6
SES								
0%– < 5% poverty	779	8.4	745	8.1	68	2.4	66	2.3
5%– < 10% poverty	1048	11	1018	11	177	6.2	167	5.9
10%– < 20% poverty	1506	16	1706	19	442	16	533	19
20–100% poverty	1085	12	1349	15	645	23	747	26
Urban/Rural								
Urban	3409	37	3609	39	1171	41	1300	46
Rural	1009	11	1209	13	161	5.7	213	7.5

^a^Percentages shown by receipt of ODX testing and characteristic within race group

**Fig. 1:**
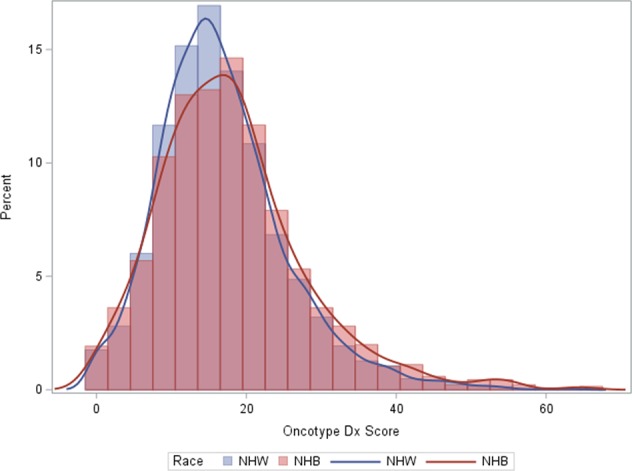
Distribution of Oncotype DX recurrence scores among non-Hispanic White (NHW) and non-Hispanic black (NHB) women diagnosed with HR + /HER2− in Georgia (2010–2014)

Overall, NHB women were more likely to present with a grade 3 breast cancer diagnosis, receive chemotherapy, be under 40 years of age, and have Medicaid health insurance at diagnosis (Table 1).^30^ NHB women were also less likely to receive a low-risk ODX RS (52% vs. 58%) and more likely to have a high-risk recurrence score (14% vs. 8.4%), but equally likely to receive an intermediate score (33% vs. 34%) (Table 1).^30^ When we applied TAILORx cutpoints a similar trend was observed, however, more women were categorized as having an intermediate recurrence score (53% NHB and 57% NHW), and less as having a high (22% NHB and 15% NHW) or low (25% NHB and 27% NHW) recurrence scores. The distribution of scores was slightly left-skewed for NHB women, suggesting a larger proportion of NHB women with higher recurrence scores (Fig. 1).^30^ Similarly, the mean and median of the ODX RS distribution was not meaningfully higher among NHB women (mean = 19.3, median = 17.0) compared to NHW women (mean = 17.3, median = 16.0).

### Receipt of chemotherapy

Per guidelines, there was variation in receipt of chemotherapy by ODX RS. Additionally, there were differences in the proportion receiving chemotherapy by race, even within strata of ODX RS. Receipt of chemotherapy was generally comparable within strata of ODX recurrence score, although NHB women with low scores were slightly more likely to receive chemotherapy (OR = 1.16, 95%CI 0.77, 1.75), and NHB women with high scores slightly less likely to receive chemotherapy (OR = 0.77, 95% CI 0.48, 1.24), than their NHW counterparts. There was no evidence of interaction between ODX RS and race on multiplicative (*p* = 0.38) or additive (RERI = −22.4, 95% CI −76.0, 31.2) scales (Table 2).^30^ Notably, among those who did not receive an ODX RS, NHB women were more likely to receive chemotherapy compared to NHW women (OR 1.33, 95% CI 1.14, 1.54) in the age and stage-adjusted model (Supplementary Table 1).^30^ Due to the uncertainty in guidelines surrounding the use of ODX RS among women with node positive disease, we repeated the analysis excluding women with limited lymph node involvement (*n* = 569). To ensure consistency of testing procedures across women, we subsequently excluded 329 women for whom we received no corresponding score from Genomic Health, Inc. (Supplementary Table 1).^30^ Results from our sensitivity analyses were comparable, although less precise.

**Table 2:** Age- and multivariable-adjusted odds ratios (OR) and 95% confidence intervals (95% CI) for receipt of chemotherapy according to race and ODX RS (ODX RS) group among non-Hispanic White (NHW) and non-Hispanic Black (NHB) women diagnosed with lymph-node-negative HR + /HER− breast cancer, an ODX RS from Genomic Health, Inc., 2010–2014 and registered with the Georgia Cancer Registry

	NHW			NHB					
ODX RS	Chemotherapy			Chemotherapy				Stratum specific	
	No	Yes	OR (95% CI)	No	Yes	OR (95% CI)	RERI^a^	OR (95% CI)^b,d^	OR (95% CI)^c,e^
Low	2438	102	Reference	634	33	1.20 (0.80, 1.80)	Reference	1.20 (0.80, 1.80)	1.16 (0.77, 1.75)
Intermediate	968	467	11.97 (9.51, 15.1)	280	168	13.4 (10.2, 17.8)	1.28 (−1.72, 4.28)	1.12 (0.89, 1.41)	1.10 (0.87, 1.38)
High	62	298	134.4 (95.1, 190)	34	155	108 (70.3, 165)	−22.4 (−76.0, 31.2)	0.80 (0.50, 1.28)	0.77 (0.48, 1.24)

^a^Relative excess risk due to interaction

^b^Age-adjusted

^c^Adjusted for age and stage

^d^*p*-value = 0.39

^e^*p*-value = 0.38

### Breast cancer-specific mortality

**Fig. 2:**
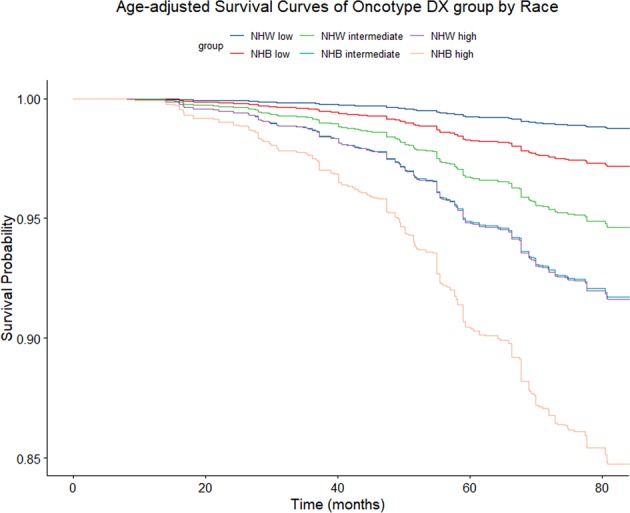
Age-adjusted survival plots by Oncotype DX recurrence risk group and race among non-Hispanic White (NHW) and non-Hispanic Black (NHB) women diagnosed with HR+/HER2− breast cancer in Georgia (2010–2014)

In the age-adjusted survival plots, the survival proportion is higher for NHW, compared to NHB, at each level of ODX RS (Fig. 2).^30^ In multivariable-adjusted models, we observed the most robust disparity among women diagnosed with a low ODX RS, whereby NHB women had 2.5 times the hazard of breast cancer mortality compared to NHW women (HR = 2.47, 95% CI 1.22, 4.99) (Table 3). This was followed by a more than two-fold disparity among women diagnosed with a high ODX RS (HR = 2.09, 95% CI 1.00, 4.37). While we observed an increase in the hazard of breast-cancer-specific mortality comparing NHB to NHW women with an intermediate ODX RS, the estimate was attenuated and included the null (HR = 1.56, 95% CI 0.93, 2.60). We did not observe evidence of multiplicative interaction between ODX RS and race on breast cancer mortality but found a slight departure on the additive scale (RERI = 4.08, 95% CI −2.27, 10.43). The results or our sensitivity analyses (excluding women with limited nodal involvement and without a score from Genomic Health, Inc.) were consistent, although hazard ratios were slightly more pronounced in the low and high ODX RS group and less precise (Supplementary Table 2).^30^

**Table 3:** Age- and stage-adjusted hazard ratios (HR) and 95% confidence intervals (95% CI) for breast-cancer-specific death according to race and Oncotype DX recurrence score (ODX RS) group among non-Hispanic White (NHW) and non-Hispanic Black (NHB) women diagnosed with HR + /HER2− breast cancer in Georgia (2010–2014)

	NHW		NHB				
ODX RS	BC mortality	HR (95% CI)	BC mortality	HR (95% CI)	RERI^a^	Stratum specific^b^	Stratum specific^c^
	Events		Events				
Low	21	Reference	13	2.57 (1.27, 5.19)	Reference	2.57 (1.22, 4.99)	2.47 (1.22, 4.99)
Intermediate	45	3.93 (2.32, 6.66)	22	6.35 (3.45, 11.68)	1.00 (−2.33, 4.32)	1.56 (0.93, 2.60)	1.56 (0.93, 2.60)
High	14	4.98 (2.51, 9.86)	15	10.82 (5.51, 21.25)	4.08 (−2.27, 10.43)	2.18 (1.05, 4.52)	2.09 (1.00, 4.37)

Multiplicative interaction *p-*value 0.55

^a^Relative excess risk due to interaction

^b^Age-adjusted

^c^Adjusted for age and stage

In an additional analysis, we explored the racial disparity in breast-cancer-specific mortality applying the TAILORx cutpoints. Due to the small number of events, we were unable to calculate an effect estimate in the low-risk group, and this group was combined with the intermediate risk group. The results of the sensitivity analysis mirrored those of the standard cutpoints, namely that in the low/intermediate risk group, we observed a two-fold increase in breast-cancer-specific mortality among NHB women compared to NHW women with the same recurrence risk score (HR = 2.10, 95% CI = 1.31, 3.37). We observed a similar, although attenuated, difference in breast cancer mortality by race in the high-risk group (HR = 1.85, 95% CI = 1.07, 3.21) (Supplementary Table 3).^30^

## Discussion

In this study, we evaluated the contribution of ODX RS as a possible mechanism to explain the racial disparity in breast cancer mortality among women diagnosed with HR+ breast cancer in Georgia. Overall, we did not observe any major race disparities in receipt of ODX RS; however, there did appear to be a one-year lag in uptake among NHB women. We did observe variation in receipt of the score among women eligible to receive the score by insurance status, socioeconomic status, and age. NHB women were less likely to be diagnosed with a low-risk recurrence score and more likely to be diagnosed with a high-risk recurrence score compared to NHW women. We identified slight racial variation in receipt of chemotherapy, wherein NHB women were more likely to receive chemotherapy if they had a low ODX RS and less likely to receive chemotherapy if they had a high ODX RS when compared to their white counterparts. When we examined racial disparities in breast cancer mortality within ODX RS groups, we observed the most pronounced disparity among the lowest recurrence score group, which constitutes the majority (~50%) of all breast carcinomas eligible for Oncotype DX testing. While we also observed racial disparities in the intermediate and high recurrence score groups, they were slightly attenuated and included the null.

Previous studies have indicated that there are racial disparities in Oncotype DX testing; however, few have been robust enough to examine variation across the breast cancer continuum (e.g., treatment and outcomes) by ODX RS group. It is well-established that NHB women are more likely to receive chemotherapy than NHW women, even after accounting for differences in patient age and tumor biology. Previous studies, including ours, have indicated that NHB women are more likely to be recommended chemotherapy and receive it than NHW counterparts.^20,23^ Some studies have shown that with Oncotype DX testing, this difference in therapy decisions is mitigated.^23^ In our study, we saw that this difference in treatment decision was mitigated in the low and intermediate recurrence score groups compared to women who did not receive ODX testing. Data generated from clinical trials, have indicated that over prescribing chemotherapy among women with low ODX RS can lead to worse outcomes and may be a contributing factor to the observed racial disparities in BC mortality in this study.^31^ Previous studies suggest that NHB women may have higher ODX RS on average compared to their white counterparts,^32,33^ which may reflect use patterns or less favorable tumor biology for ER-positive disease. Oncotype DX was developed in a primarily homogenous NHW population, although with demonstrated utility in the clinical setting, it has not necessarily been validated in an exclusively NHB population, given the persistent underrepresentation of minority groups in clinical trials.^34,35^ Thus, observational studies can serve to validate clinical results in more diverse population groups, thereby enhancing the generalizability and transportability of the trial results.^36–39^ In our current study, we found slight departures in survivorship between NHB and NHW women within strata of ODX RS; however, the most pronounced differences were among those with the lowest recurrence score. Most of these women would not receive chemotherapy based on the Oncotype DX recommendations but would receive endocrine therapy, therefore the observed racial disparity in this subgroup may be driven by adherence to endocrine therapy, especially over the long term. Previous studies have reported that black women are more likely to report non-adherence and discontinuation of endocrine therapy compared to NHW women.^40,41^ Early discontinuation and non-adherence to adjuvant endocrine therapy are associated with adverse breast cancer outcomes.^42^Therefore, future research would benefit from studies that investigate this relationship further. Results presented in the 2018 AACR *San Antonio Breast Cancer Conference* on clinical outcomes in breast cancer and race from the TAILORx trial, indicated that NHB women had worse clinical outcomes and a higher risk of recurrence despite similar 21-gene assay recurrence score results and adjuvant therapy.^43^ This may suggest that the genes selected in the ODX RS may not be relevant to this population women and some proportion of NHB women with a low ODX RS may benefit from chemotherapy, although authors reported that TAILORx did not demonstrate a differential chemotherapy benefit in NHB women with an intermediate ODX RS (11–25), despite worse outcomes.

Our study has many strengths, namely that it leverages resources from a high-quality population-based registry among a well-defined source population with access to gene expression profiling from Genomic Health. Using this information rich data source, we were able to examine ODX RS in their contribution to variation in receipt of chemotherapy and breast-cancer-specific mortality. While the end-point used in this analysis was mortality, it is an imperfect proxy of recurrence. ODX RS is a measure of recurrence risk, which is not routinely collected in state or national cancer registries. Further, the follow-up time was relatively short (mean follow-up of ~5 years), which limited the number of events that we observed within strata of ODX RS. Given that our study focuses on women with HR+disease, 10-year risk of recurrence or breast cancer mortality may be important to understand race differences in recurrence scores. There are additional limitations in the use of the ODX RS from Genomic Health and the GCR. Genomic Health only provides registries with recurrence scores for women having tumors that are indicated for an ODX RS (HR + , HER2− per RT-PRC), so we used the presence of an ODX RS from Genomic Health as the gold standard, but also included Oncotype DX scores that were recorded in the registry if the patient was indicated as being eligible to receive an Oncotype DX score based on NCCN guidelines. These differences may be due to possible misclassification bias, where the eligibility criteria from the registry may be misclassified. It is also possible that the ODX RS recorded in the GCR was misclassified. Nonetheless, our sensitivity analyses suggest this bias is minimal. Additional validation of ODX RS captured by registries are necessary to ensure accuracy in their use in epidemiologic studies.^44,45^ Receipt of chemotherapy is under ascertained in cancer registry data, with an estimated 69% sensitivity when records were compared to Medicare claims.^46^ Finally, registry data are missing information on adherence to endocrine therapy, type and completion of chemotherapy, and comorbidities at diagnosis, which may be important covariates affecting chemotherapy indication and breast cancer outcomes.

In conclusion, in this study we observed variation in receipt of chemotherapy by ODX RS and race, wherein NHB women were less likely to receive chemotherapy within the highest ODX RS group. We observed the highest disparity in breast-cancer-specific mortality among women in the lowest ODX RS group, although the association was fairly constant in the intermediate and high recurrence score groups. Further research into understanding the downstream factors, such as receipt of guideline care, adherence to endocrine therapy, and treatment delay, may be important drivers of the disparity in this otherwise prognostic favorable subtype of tumors. Additional validation of the 21-gene assay among NHB and further identification of prognostic biomarkers unique to specific racial/ethnic subgroups may also be warranted.

## Methods

### Study population

**Fig. 3:**
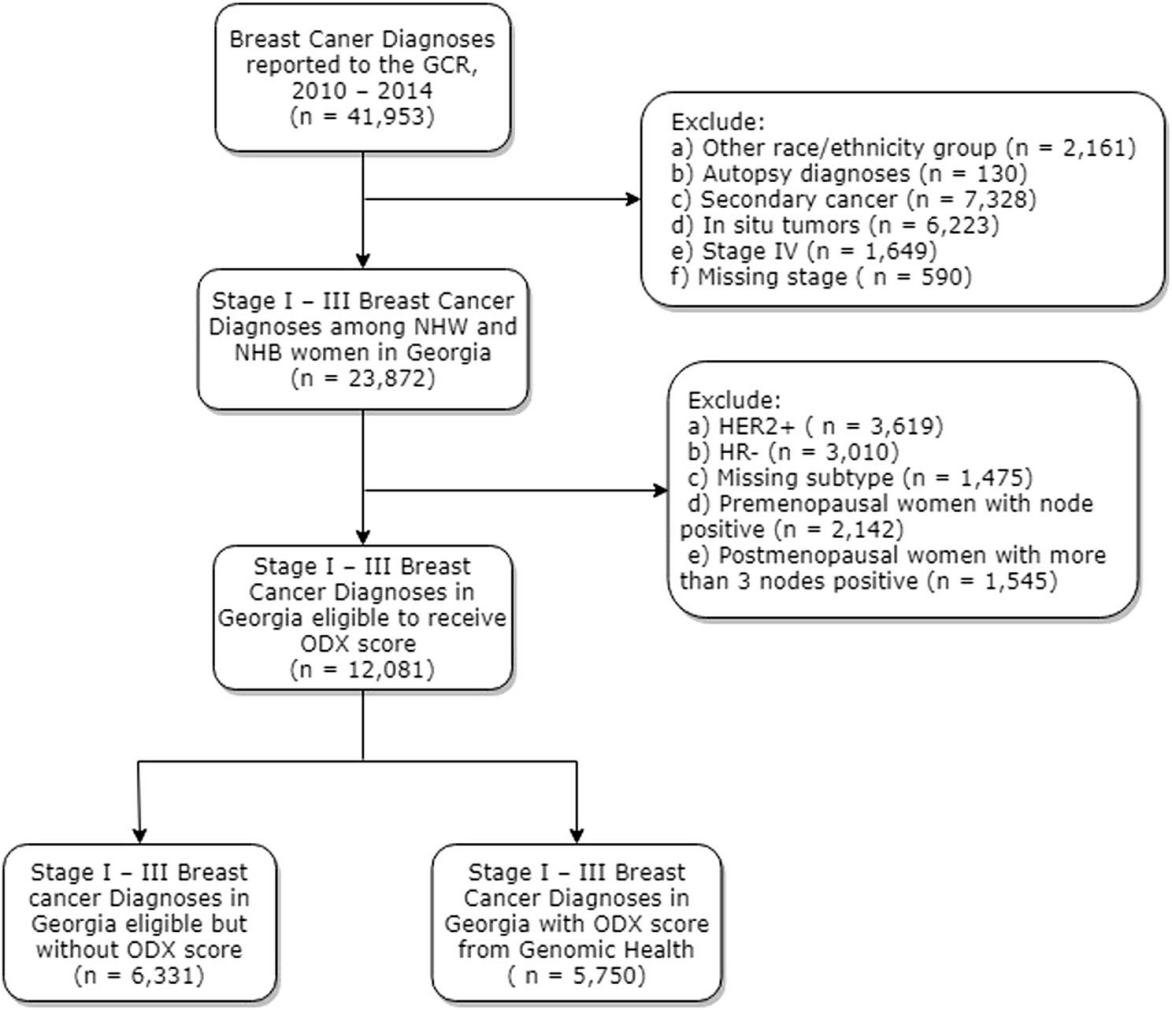
Identification of the eligible study population using the Georgia Cancer Registry (GCR) resources (2010–2014)

The GCR is a statewide population-based registry that has collected nearly all cancer cases diagnosed among Georgia residents since 1 January 1995, informing cancer prevention and control activities for the state. Using this registry, we identified breast cancer diagnoses (ICD-O-3 = C50) among Georgia residents that occurred between 1 January 2010 and 31 December 2014. Patients were included if they were classified as being NHB or NHW with a stage I–III first primary breast cancer at diagnosis. Additionally, we restricted to women who were indicated for an ODX RS— women with an HR-positive, HER2-negative, and node-negative or postmenopausal (≥55 years of age) and node positive (1–3 LN+), breast cancer. We then identified women with a verified Oncotype DX^®^ recurrence score. Race and ethnicity were obtained from documentation in medical records using classification similar to the 2010 census, when available. Hispanic ethnicity was determined by the NAACCR Hispanic Identification Algorithm (NHIA), which uses a combination of standard variables to classify cases as Hispanic or non-Hispanic for analytic purposes.^47^ All other diagnoses were excluded, including those of other race/ethnic groups, diagnoses verified through autopsy, missing stage, stage 0 and IV breast carcinomas, and any secondary/multiple primary tumor diagnoses (Fig. 3).^30^ This study was conducted in accordance with the Declaration of Helsinki and has been approved by the institutional review board (IRB) of Emory University (IRB00099875) on 24 October 2017. Participant consent was not required due to the registry-based nature of the study.

### Exposure assessment

Results from the 21-gene Oncotype DX Breast Recurrence Score assay (Genomic Health, Inc., Redwood City, CA, USA) are available to the Georgia Cancer Registry. As part of the NCI’s Surveillance, Epidemiology and End Results (SEER) Program, SEER registries link their data with Genomic Health on a scheduled basis to obtain Oncotype results. Genomic Health provides results after additionally clarifying that patients are indicated for receipt of ODX RS (HR +, HER− per RT-PCR). We used the scores available from Genomic Health for all breast cancer patients identified as being eligible to receive on ODX RS the purposes of this study. We also included scores from GCR if there was no score from Genomic Health Inc to enhance study data (*n* = 329). For breast cancer patients with scores both from Genomic Health Inc and recorded in the GCR, we used scores from Genomic Health Inc. in the case of any discrepancies. In general, there was high concordance between scores recorded in the GCR and those from Genomic Health, Inc. (80%) (Supplementary Table 4).^30^ The current guidelines categorize the 21-gene recurrence score into low [<18], intermediate [18–30], and high [≥31] risk groups, which were used in this analysis. Results from the recently published TAILORx trial defined recurrence score risk groups as: low [<11], intermediate [11–25], and high [>25], which were used in an additional set of exploratory analyses.

### Outcome assessment

Receipt of chemotherapy was determined based on first course of treatment as recorded in the GCR. Chemotherapy was considered not to have been administered if it was not part of the treatment plan, or if it was not administered due to patient refusal or contraindications for chemotherapy. Chemotherapy was considered to have been administered if it was documented in the medical records as indicated for first course therapy. If it was noted that chemotherapy was unknown to have been administered, then it was considered to be missing in our dataset.

The underlying cause of death was determined from the death certificate with valid ICD-10 codes. The GCR routinely links to state death certificates to identify deaths and causes of death from the preceding year. Additionally, the GCR annually links to the US National Death Index to identify deaths that occur outside of Georgia. In this study, we included all deaths recorded up until 31 December 2016 collected by the US National Death Index, and all deaths in Georgia until 31 December 2018 collected by the Georgia Office of Vital Records. An estimated 6% of breast cancer deaths from women diagnosed in Georgia occur outside of the state.

### Covariates of interest

Tumor characteristics from the registry used in the determination of the study population and analysis included cancer stage at diagnosis, tumor grade, expression of the estrogen receptor (ER), expression of progesterone receptor (PR), and molecular subtype. Cancer stage at diagnosis was based on individual variables captured by the registry that allow the derivation of a combined clinical/pathologic stage group using the same definitions as the AJCC 7th edition staging manual. Tumor grade was categorized as 1, 2, or 3+. Hormone receptor (HR) expression was classified as positive or negative based on the expression of either ER or PR. If ER or PR were classified as borderline, then HR expression was considered positive; however, if both ER and PR were borderline then HR status was considered unknown/missing.

We considered different patient demographic characteristics present at the time of diagnosis that may contribute to differences in uptake of the 21-gene ODX RS. This included type of health insurance (uninsured, private, Medicaid, and Medicare), age at diagnosis (<40, 40–49, 50–65, >65 years of age), urban/rural residence, area-based residential socioeconomic status (SES) index based on the percent of the census tract population below the federal poverty level (0%–<5%, 5%–<10%, 10%–<20%, 20–100%). Patient demographic information was abstracted from the GCR.

### Statistical methods

Descriptive statistics were calculated as median values with interquartile ranges, or frequency and percent for covariates across receipt of ODX RS and population subgroup. Follow-up was defined as time in months, from the date of diagnosis until the first of (a) mortality event, (b) last date of contact in the registry, or (c) 31 December 2018. We used age-adjusted and multivariable-adjusted logistic regression to calculate the odds ratios (OR) and 95% confidence intervals (CI) for the odds of chemotherapy receipt by self-identified race and Oncotype DX recurrence group. Due to uncertainty in the guidelines for using ODX among women with positive lymph nodes, we stratified our analyses by lymph node positivity, reporting primary results among women with no lymph node involvement and including those with lymph node positivity in a supplementary table. Secondly, we used age-adjusted and multivariable-adjusted Cox proportional hazard models to estimate the hazard ratios (HR) and 95% confidence intervals (CIs) for the association between self-identified race and ODX RS on breast-cancer-specific mortality. We verified the proportional hazards assumption for each covariate using ln-ln survival curves and an interaction term of the covariate with time. To assess the presence of interaction between race and the ODX RS, we used the common referent approach to calculate the relative excess risk due to interaction (RERI) to evaluate departure of the effect on the additive scale, RERI = OR_11_ − OR_10_ − OR_01_ + 1 and RERI = HR_11_ − HR_10_ − HR_01_ + 1.^48,49^ We used the delta method to calculate the 95%CI for the RERI using the variance-covariance matrix of the effect estimates. We evaluated the presence of multiplicative interaction using the likelihood ratio test with the presence of interaction terms in the model and reporting the stratum specific effect estimates. Based on a priori knowledge and in our graphical assessment (DAG),^50^ we did not identify any other variables besides age or stage to be potential confounders in the association for receipt of chemotherapy or breast-cancer-specific mortality. In our exploratory analysis using the TAILORx cutpoints, we combined the low and intermediate recurrence score groups due to the small number of events (<4). All analyses were carried out using R and SAS v9.4 (Cary, NC).

### Reporting summary

Further information on research design is available in the Nature Research Reporting Summary linked to this article.

## Supplementary information

Supplemental Document

NR Reporting summary

## Data Availability

The data generated and analysed during this study are described in the following data record: 10.6084/m9.figshare.9674996.^30^ The data supporting the figures and tables in this published article are not publicly available to protect patient privacy, but can be accessed from the corresponding author on reasonable request. Data will be made available from the Georgia Center for Cancer Statistics at Emory University to authorized researchers who have an approved institutional IRB application and have obtained approval from the Georgia Department of Public Health IRB committee. Please email: dph-datarequest@dph.ga.gov for access to the data.

## Code availability

Relevant computer code is available upon request to the corresponding author (Lindsay J Collin).
