# Supplementary material for: Oncotype DX recurrence score implications for disparities in chemotherapy and breast cancer mortality in Georgia
Source: NPJ Breast Cancer. 2019 Sep 26;5:32. doi: 10.1038/s41523-019-0129-3 (PMC6763428; doi:10.1038/s41523-019-0129-3)
Supplement: Supplementary file 1 — Supplemental Document [file 41523_2019_129_MOESM1_ESM.pdf]

Oncotype DX recurrence score implications for disparities in chemotherapy and breast cancer mortality in Georgia

Lindsay J. Collin,[1] Ming Yan[1], Renjian Jiang [1], Kevin C Ward[1], Brittany Crawford[2], Mylin A Torres[3,4], Keerthi Gogineni[5], Preeti D Subhedar[6], Samantha Puvanesarajah[7], Mia M Gaudet[7], Lauren E McCullough[1]

Supplementary Table 1. Multivariable-adjusted odds ratios (OR) and 95% confidence intervals (95% CI) for receipt of chemotherapy according to race and Oncotype DX recurrence score (ODX RS) group among non-Hispanic White (NHW) and non-Hispanic Black (NHB) women diagnosed with breast cancer, an Oncotype DX recurrence score from Genomic Health, Inc., 2010–2014 and registered with the Georgia Cancer Registry, excluding those with 1) lymph node positive (1–3 LN+) breast cancer and 2) an ODX RS from GCR and no corresponding score from Genomic Health.

	Chemotherapy					
	NHW		NHB			
**ODX RS**	**No**	**Yes**	**No**	**Yes**	**Exclude LN+ 1–3 (n=569)**	**Exclude ODX RS from GCR (n=329)**
**Low**	2212	64	580	20	1.08 (0.65, 1.81)	0.96 (0.55, 1.67)
**Intermediate**	887	419	256	146	1.04 (0.81, 1.33)	1.01 (0.79, 1.31)
**High**	54	279	32	143	0.69 (0.42, 1.14)	0.61 (0.33, 1.13)
					**Age and stage-adjusted**	
**No Score**	3251	1235	877	600	1.33 (1.14, 1.54)	

Supplementary Table 2. Multivariable-adjusted hazard ratios (HR) and 95% confidence intervals (95% CI) for breast cancer specific death according to race and Oncotype DX recurrence score (ODX RS) group among non-Hispanic White (NHW) and non-Hispanic Black (NHB) women diagnosed with breast cancer with an Oncotype DX recurrence score from Genomic Health Inc., 2010–2014 and registered with the Georgia Cancer Registry, excluding those with 1) lymph node positive (1–3 LN+) breast cancer and 2) an ODX RS from GCR and no corresponding score from Genomic Health.

ODX RS	NHW Events	NHB Events	Exclude LN+ 1–3 (n=569)	Exclude ODX RS from GCR (n=329)
**Low**	16	12	2.96 (1.38, 6.37)	3.11 (1.38, 6.99)
**Intermediate**	39	17	1.40 (0.79, 2.48)	1.43 (0.80, 2.54)
**High**	14	15	1.94 (0.92, 4.09)	2.48 (1.07, 5.76)

Supplementary Table 3. Age-adjusted and multivariable-adjusted hazard ratios (HR) and 95% confidence intervals (95% CI) for breast cancer specific death according to race and Oncotype DX recurrence score (ODX RS) group using TAILORx cutpoints among non-Hispanic White (NHW) and non-Hispanic Black (NHB) women diagnosed with breast cancer with an Oncotype DX recurrence score, 2010–2014 and registered with the Georgia Cancer Registry (n=5750).

	NHW		NHB			Stratum Specific	
**ODX RS**	**BC mortality**	**HR (95% CI)**	**BC mortality**	**HR (95% CI)**	**RERI (95% CI)**	**Age-adjusted**	**Age and stage adjusted**
**Low/ Intermediate**	50	reference	27	2.08 (1.29, 3.33)	reference	2.08 (1.29, 3.33)	2.10 (1.31, 3.37)
**High**	30	3.52 (2.23, 5.55)	23	6.32 (3.84, 10.43)	1.73 (-1.28, 4.74)	1.80 (1.04, 3.11)	1.85 (1.07, 3.21)
						P -interaction = 0.69568	*P - interaction = 0.73121*

Supplementary Table 4: Agreement between Oncotype DX recurrence scores from the Georgia Cancer Registry and Genomic Health Inc., among non-Hispanic White and non-Hispanic Black women diagnosed with a HR+/HER2– breast cancer in Georgia (2010–2014).

	Genomic Health			
**Georgia Cancer Registry**	**Missing**	**Low**	**Intermediate**	**High**
**Missing**	6331	867	483	102
**Low**	109	2149	16	0
**Intermediate**	60	11	1294	6
**High**	160	130	72	291
